# High-Throughput Whole-Exome Sequencing and Large-Scale Computational Analysis to Identify the Genetic Biomarkers to Predict the Vedolizumab Response Status in Inflammatory Bowel Disease Patients from Saudi Arabia

**DOI:** 10.3390/biomedicines13020459

**Published:** 2025-02-13

**Authors:** Hanin Aljohani, Doaa Anbarserry, Mahmoud Mosli, Amani Ujaimi, Duaa Bakhshwin, Ramu Elango, Sameer Alharthi

**Affiliations:** 1 Department of Clinical Pharmacology, Faculty of Medicine, King Abdulaziz University, Jeddah 22252, Saudi Arabia; hsaleamaljohani@stu.kau.edu.sa (H.A.); dserri@kau.edu.sa (D.A.); dbakhshwin@kau.edu.sa (D.B.); 2 Department of Medicine, Faculty of Medicine, King Abdulaziz University, Jeddah 22252, Saudi Arabia; mmosli@kau.edu.sa; 3 Inflammatory Bowel Disease Research Group, King Abdulaziz University, Jeddah 22252, Saudi Arabia; 4 Inflammatory Bowel Disease Unit, King Abdulaziz University Hospital, King Abdulaziz University, Jeddah 22252, Saudi Arabia; 5 Princess Al-Jawahara Cernter of Excellence in Research of Hereditary Disorders (PACER-HD), King Abdulaziz University, Jeddah 22252, Saudi Arabia; aaujaimi@gmail.com; 6 Department of Genetic Medicine, Faculty of Medicine, King Abdulaziz University, Jeddah 22252, Saudi Arabia

**Keywords:** vedolizumab, genetic biomarkers, ulcerative colitis, Crohn’s disease, drug response, whole-exome sequencing, rare variants

## Abstract

**Background/Objectives**: Vedolizumab (VDZ) is the new monoclonal drug targeting α4β7 integrin for patients with moderate/severe IBD. Between 30 and 45% of patients fail to respond to VDZ after 14–16 weeks of treatment. The aim of the study was to explore the genetic profile of vedolizumab-treated Arab IBD patients in Saudi Arabia to identify the potential biomarkers to differentiate the responders from non-responders. **Methods**: A cohort of 16 patients with IBD, including 4 with Crohn’s disease and 12 with ulcerative colitis, were recruited. Following 16 weeks of VDZ treatment, nine were found to be responders and seven non-responders. Blood samples were collected for the whole exome sequencing of DNA from all patients. The variants in the whole-exome sequencing data were analyzed with a variety of bioinformatics tools and databases, such as Polyphen2, Mutation Taster, CADD, FATHMM, Open Target Platform, TOPPFun, STRING, and GTEx. **Results**: More than 1.6 million variants from 16 samples were analyzed. The rare variant analysis prioritized NOD2, IL23, IL10, IL27, and TRAF1 genes in non-responders. NOD2, IL23, IL10, IL27, and TRAF1 were found to be the significant IBD risk factors in multiple genome-wide association studies, and their pro-inflammatory activity might contribute to the inherent resistance to VDZ. Rare variants of CARD9, TYK2, IL4, and NLRP1 genes present in VDZ responders enhance the anti-inflammatory/immune modulation effects. **Conclusions**: This investigation is the first to apply whole-exome sequencing to identify the potential drug response biomarkers for the IBD drug VDZ in Saudi Arabia.

## 1. Introduction

Inflammatory bowel disease (IBD) is a persistent and progressive disease of the digestive tract, caused by an inappropriate immune system response to microorganisms [1]. IBD is a broad term that is classified into two major forms, Crohn’s disease (CD) and ulcerative colitis (UC), depending on their specific anatomical sites [2]. It is estimated that there are approximately 1.5 million IBD patients in the United States, 2.2 million in Europe, and a substantial number worldwide [3]. In Saudi Arabia, the epidemiology of IBD remains unknown; however, several studies in various regions suggest an increase in incidence over the years, ranging from 0.32 per 100,000 to 1.66 per 100,000 per year [4,5,6].

Over the last two decades, different highly promising therapies have emerged for autoimmune and inflammatory conditions such as IBD. However, a major challenge is that not all patients respond to these treatments [7]. Approximately 30% of IBD patients do not respond to anti-TNF therapy (PNR: primary non-response), and nearly half of patients who respond to anti-TNF therapy lose clinical benefit within the first year, requiring dose escalation or treatment modification (LOS: secondary loss of response) [8,9]. PNR and LOS result in unsuccessful disease control and the exacerbation of symptoms [10]. Between 10 and 40 percent of patients exhibited PNR to anti-TNF medications after the initial treatment period of 14 weeks, while an additional 24 to 46 percent suffered LOS after one year of anti-TNF medication [11,12,13]. A new monoclonal antibody that blocks the activity of integrin alpha 4 beta 7 was developed in 2014. Vedolizumab (VDZ) is a monoclonal antibody that prevents the movement of white blood cells in the intestine by disrupting the connection between a protein called integrin alpha 4 beta 7 and mucosal vascular addressin cell adhesion molecule 1 (MADCAM-1). A significant association between clinical remission at week 14 and median VDZ value at week 6 (25.1 μg/mL 95% confidence interval: 16.5–42.9), compared to non-responders, was reported from Saudi Arabia [14].

A combination of multiple factors, including environmental, genetic, immune, and gut microbiota factors, leads to an abnormal immune system. This defect triggers the production of inflammatory cytokines and adhesion molecules, which initiate the inflammatory process [15]. A few reports indicated a connection between immune cell phenotype and the effectiveness of VDZ. The pathogenesis of IBD involves the participation of both innate and adaptive immune cells, including CD4+ helper T cells and macrophages in the patient’s colonic mucosa.

A lack of biomarkers to predict the non-responders to various drugs early leads to the persistence and severity of symptoms in IBD patients. Next-generation sequencing (whole-exome and whole-genome sequencing) and the large-scale generation of variants in populations are used to identify the causal or contributing genes and variants for IBD [16,17,18]. The same technologies can be used to identify biomarkers at the molecular level (rare variants with minor allele frequency <0.05) for drug response status. Multiple studies have documented pharmacological parameters, such as peak and trough level, predicting the effectiveness of VDZ [14,19]. The GWASs (genome-wide association studies) were mainly used to identify 100s of disease susceptibility loci but not the drug response biomarkers in IBD so far. The current study is the first to apply whole-exome sequencing to study the complex disease drug response in the Saudi Arabian population, marking a unique ethnic-specific contribution. It assesses the exomes, bringing to light rare but significant genetic variations relevant to IBD drug response. The discovery and validation of these biomarkers could optimize treatment effectiveness and pave the way for more personalized treatment plans for IBD patients.

The aim of the study was to explore the genetic profile of the vedolizumab-treated IBD patients to identify the potential biomarkers for responders among Arabs of Saudi Arabia.

## 2. Materials and Methods

This study is one part of research for which Institutional Research Ethics Board approval was obtained prior to participant recruitment and sample collection (KAUH-REC No. 480-20) [14]. Patients were diagnosed with IBD by gastroenterologists at King Abdul-Aziz University Hospital in Jeddah, Saudi Arabia. Informed consent was obtained from all participants prior to collecting clinical data and blood samples during the study phase. Blood samples were collected in EDTA vials for DNA analysis and stored at −80 °C till they were used.

1. Participant profile:

The following Table 1 presents the inclusion/exclusion criteria used to identify and recruit the participants for the study.

**Figure 1:**
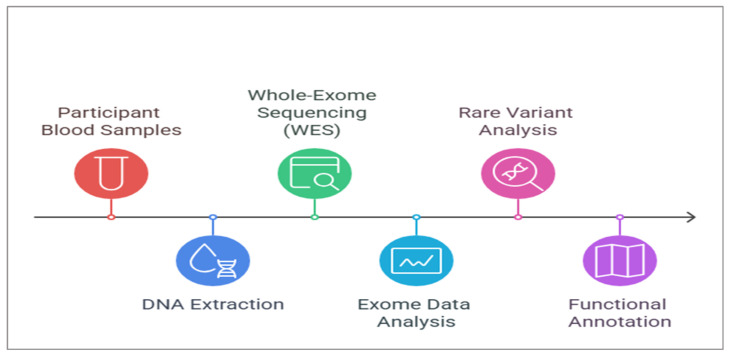
General workflow of the study.

The data collection form in Supplementary Materials: Research information comprises several items, including clinical and family history and physical examination with the clinical scoring. All patients who started vedolizumab treatment between October 2020 and September 2021 were identified and offered the option to join the research project. Of the 25 patients who fulfilled the criteria as VDZ-naïve IBD patients, only 16 joined and signed the informed consent form for data and sample collection. At weeks 0, 2, and 6, patients received vedolizumab intravenous infusion (300 mg) for the induction phase and underwent a follow-up assessment at week 14. After 14 weeks of VDZ treatment, the clinical outcome was evaluated with partial Mayo score for UC and CD activity and Harvey Bradshaw scores for CD, which was confirmed by the significant VDZ trough level differences (25.1 µg/mL 95% CI: 16.5–42.9 for responders and 7.7 µg/mL, 95% CI: 4.6–10.6 for non-responders; *p* = 0.002).The general study workflow is presented in Figure 1.

**Figure 2:**
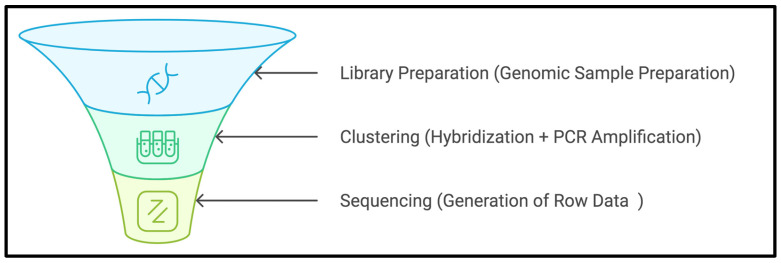
WES general workflow.

2. Exome sequence generation:

WES: DNA extraction: About 2–4 mL of venous blood samples were collected once during the study period for DNA isolation and extractions using the standard DNA extraction procedures of the lab. Quality control of DNA: The quantity of DNA was measured by the picogreen method using Victor 3 fluorometry, and again by fluorescence-based quantification for WES library preparation. Assessing the nature of the DNA: The assessment was carried out by the agarose gel electrophoresis method. DNA fragments <1 kb: A 2100 Bioanalyzer was used to check the size, and the Femto-Pulse method was used to fragment the high-molecular DNA. The Exome Capture Library from Agilent (version 7) was used to capture the targeted exome fragments, amplified by PCR. After the purification of captured fragments, extensive QC checks were carried out. Once the samples passed the QC check, the samples were loaded into the NGS machine for sequence generation for downstream analysis, as illustrated in Figure 2.

**Figure 3:**
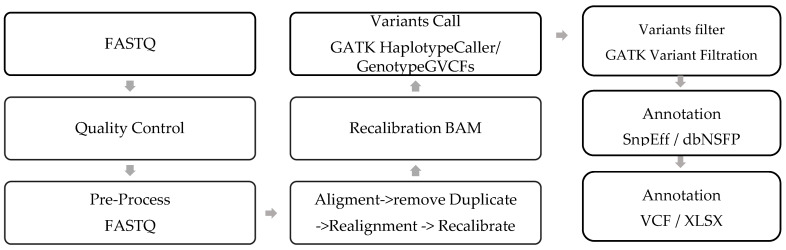
Exome sequence data analysis process and workflow.

3. The following chart provides the exome variant analysis workflow from Raw Data generated by the NGS machine to the annotated variants, (Figure 2 and Figure 3). Table 2 and Table 3 lists the various tools and databases (with versions) used for WES data analysis and variant annotations.

For variant analysis and selection, pathogenicity prediction, sequence conservation, and variant location with reference to genes, many tools were used: Poyphen2, Mutation Taster, CADD, FATHMM, etc. Only variants located in the coding region were selected for downstream analysis. Population frequency data of variants in 1K genome, ExAC, gnomAD, and RGC Million Exome variants databases were extensively used to select the rare variants with MAF <0.05 in different ethnic groups. The filtered variants from responder and non-responder groups were classified with three d levels of rare minor allele frequency (MAF) categories in all ethnic populations of the 1K, ExAC, and gnomAD databases. The minor allele frequency categories selected are (a) rare: 3 to 5% (0.03–0.05), (b) very rare: 1 to 2.99% (0.01–0.0299), and (c) extremely rare: less than 1% (<0.01).

In order to prioritize the genes and variants and to explore the Molecular Functions, Biological Processes, Cellular Components, pathways, drug targets, and the causal natures of the genes, multiple bioinformatic tools and databases were extensively used. For biological functional and pathway analysis, some of the tools and databases used are TOPPFun, Open Target Platform, KEGG, Reactome, STRING db, GTEX, etc. (Table 4).

## 3. Results

### 3.1. Participant Profile

Sixteen (sixteen out of twenty-five) IBD participants fulfilled the inclusion and exclusion criteria, including four with CD and twelve with UC. Others did not agree to join the research project and were not included in the study. The average age of the nine male and seven female patients in this study was 30 years. After 14 weeks of VDZ treatment, the clinical outcome was assessed by partial Mayo score for UC and CD activity and Harvey Bradshaw scores for CD, which was confirmed, but with significant differences in VDZ trough levels (25.1 μg/mL, 95% confidence interval: 16.5–42.9 for responders and 7.7 μg/mL, 95% confidence interval: 4.6–10.6, *p* = 0.002). Nine were classified as responders (R group), and seven were non-responders (NR group), for VDZ based on the above-mentioned internationally accepted standards by the clinical team.

### 3.2. WES Analysis

On average, 99.99% of the exome library fragments passed the quality score of Phred 30 from the target regions. Sequences that passed the Phred Q score were processed for downstream analysis. An average of 60,447,882 reads for each sample of DNA was generated, with an average sequence size of 140 bp and an average throughput depth of 198.5; the mean depth of the target regions was 96.7. A total of 1.7 million variants were identified in 16 samples with ~120 K variants per exome.

### 3.3. Stringent Variant Prioritization

The pooled responder group (nine participants) had 2590, 3308, and 9276 extremely rare, very rare, and rare variants, respectively. For the NR group (seven participants), 1808, 2421, and 5839 extremely rare, very rare, and rare variants were identified and pooled, respectively (Table 5).

**Figure 4:**
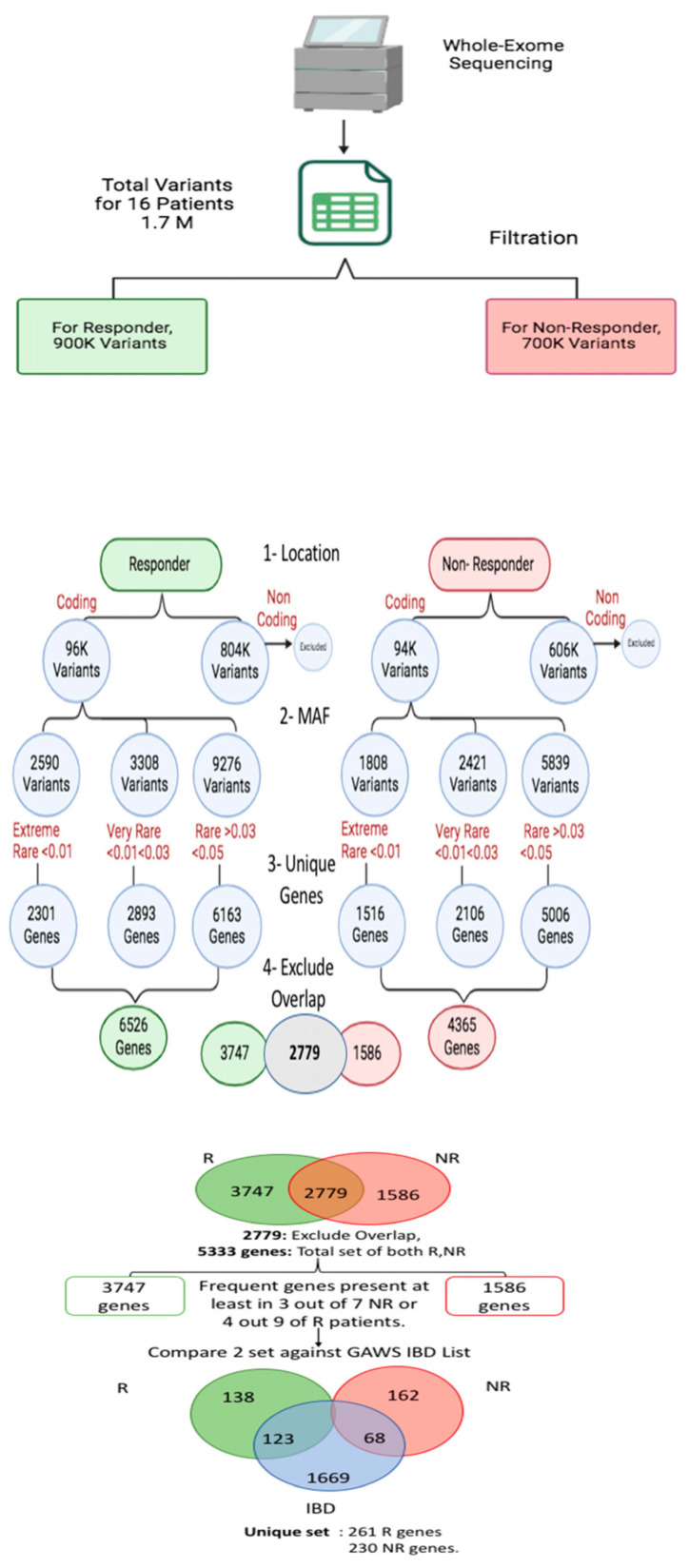
WES data workflow of variants to prioritized genes from responders and non-responders to VDZ in Saudi Arabian patients.

Prioritized variants were mapped to genes for R and NR groups, and the overlapped ones were excluded from the downstream analysis (Figure 4). The IBD GWAS data from the GWAS catalogue were compared with R and NR unique genes to find the interesting potential candidate genes.

#### 3.3.1. Prioritization Candidate Genes for R (Responder) and NR (Non-Responder) Groups

Figure 3 shows 123 and 68 genes in responder and non-responder groups’ overlaps with IBD loci. A search for functional annotation support for both sets of genes for their role in IBD development and/or autoimmunity from Open Target Platform (OTP) and TOPPFun was performed. Finally, 25 and 18 from R (from 6526) and NR (from 4365 genes) groups, respectively, were prioritized using various stringent genetic, genomic, and functional criteria as strong potential candidate biomarkers. Gene ontology: Of the prioritized 25 responder genes, 4 could affect the immune aspect through positive regulation of the adaptive immune response and NF-kb transcription factor activity as biological processes, whereas, of the 18 NR genes, 5 were enriched with the positive regulation of cytokine secretion and response and mediated IL-12 production.

##### Functional Enrichment

**Figure 5:**
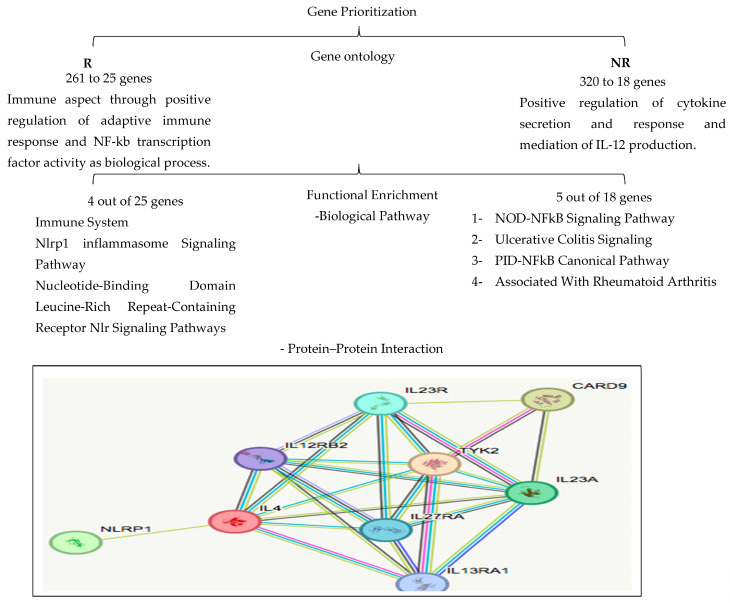
Network of responder genes (STRING protein–protein interaction dataset).

**Figure 6:**
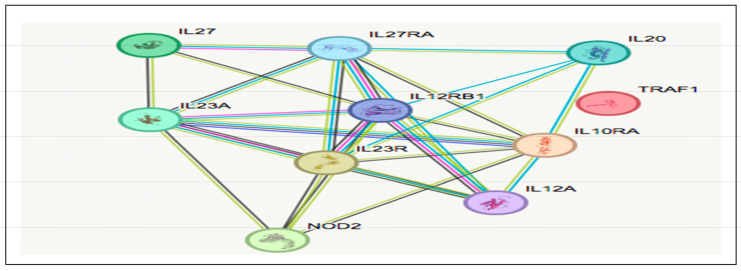
Network of genes from non-responders (STRING protein–protein interaction database).

**Figure 7:**
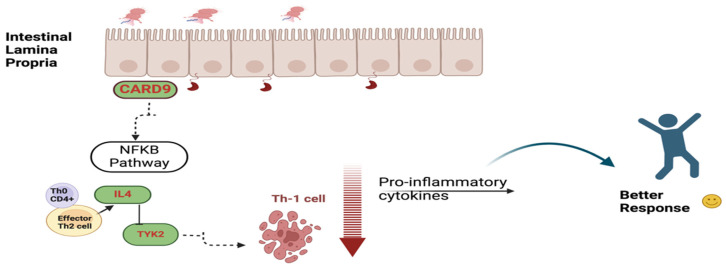
Biological functional roles of candidate genes in VDZ responder group.

- Biological pathways: Four responder candidate genes (CARD9, NLRP1, IL-4, and TYK2) may enhance the action of VDZ. These genes play an important role in one or multiple functions and pathways. These include the anti-inflammatory effect, impairment of the activation of NFkB by decreased secretion of cytokines by innate immune cells, induction of Th-2 cells to produce anti-inflammatory and immunomodulatory factors, and activation of the JAK/STAT pathway. These are involved in the following pathways: (a) immune system, (b) NLRP1 inflammasome signaling, (c) nucleotide-binding domain leucine-rich repeat-containing receptor NLR signaling, and (d) interleukin-10 signaling. Table 6 lists the key biological functions of these key genes, which potentially enhance the response to VDZ.
- The five non-responder (NR) candidate genes (NOD2, TRAF1, IL10RA, IL-23R, and IL27) enhance the activation of pro-inflammatory cytokines through MAPK and NFkB pathways, modulate innate and acquired immunity, and activate Th-17 cells, leading to the production of pro-inflammatory cytokines. These functions might contribute to the poor response to VDZ in IBD patients through the following pathways: (a) NOD-NFkB signaling, (b) ulcerative colitis signaling, and (c) the PID-NFkB canonical pathway. Those genes were also found to be associated with the development of the other common autoimmune disease, rheumatoid arthritis. Table 7 highlights the main biological functions of NR genes possibly contributing to poor or non-responder status to VDZ. The activation of pro-inflammatory cytokines through NFκB pathways by NOD2 and TRAF and modulation of immunity by IL10RA and IL27 genes was found.
- Protein–protein Interaction: The STRING protein–protein interaction database search resulted in findings of critical gene networks for both R and NR sets of genes, Figure 5 and Figure 6. The R group gene network was enriched with the cytokine processes and pathways, which include IL13RA1 and IL-12RB2. They play a crucial role with IL-4 in the anti-inflammatory process. The strong interaction between the four responder group genes highlights their potential contribution to the positive response to VDZ.
- Figure 7 explains the role of CARD9 in inhibiting the NFkB pathway, which leads to the suppression of the Th-1 cell, which is responsible for producing pro-inflammatory cytokines. They inhibit cytokine secretion. Moreover, Th-1 cells inhibited by IL-4 and TYK2 suppress the production of cytokines.

**Table 6:** Major biological role of the potential candidate gene in the VDZ responders group.

Gene Name	Function
CARD9	Impairs the activation of ReIB, a subunit of non-canonical NFkB, ↓cytokines and chemokines production by innate immune cells.
IL4	Anti-inflammatory and immunomodulatory factor, induces Th2 cell differentiation and expression.
NLRP1	Innate immune sensor to attenuate IL18, IL-1β levels.
TYK2	Activation of the Janus kinase/transcriptional signaling and activation factor (JAK/STAT) pathway.

**Table 7:** Major biological roles of the potential candidate genes in VDZ non-responders.

Gene Name	Function
NOD2	Activation of pro-inflammatory cytokines and regulation of inflammation and cell apoptosis via the MAPK and NF-κB pathways.
IL10RA	Regulates both innate and acquired immunity.
IL23R	Activation and development of the Th17 lineage and its effect on dendritic cells and macrophages results in the production of a variety of pro-inflammatory molecules.
IL27	Th17 differentiation and immune regulation.
TRAF1	Canonical and non-canonical NFκB pathways activating MAP kinases.

**Figure 8:**
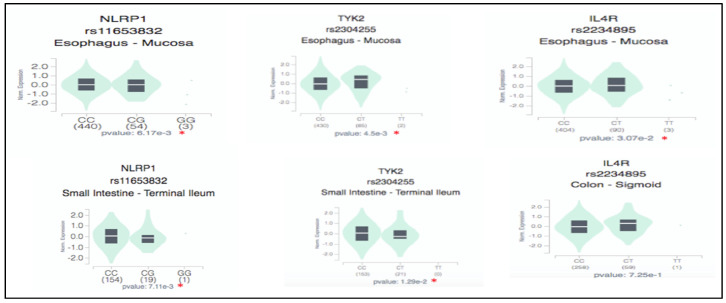
Genotype-Tissue Expression (GTEx) of genes in the responder group in different tissues. * denotes statistically significantly different gene expression recorded in specific tissue between the carriers of the rare variant and common alleles.

To further validate the biological pathways of the listed genes in the responder group, the GTEx database was explored regarding different tissues for changes in the expression of samples with variants and normal alleles. The GTEx analysis revealed a significant change in the expression of the rare variant of the IL-4R gene (rs2234895) in the esophagus; *p* = 3.07^−2^. Additionally, the TYK2 gene variant (rs2304255) showed significant expression in both the esophagus and small intestine (*p* = 4.5^−4^ and 1.29^−2^, respectively) when compared to the normal allele carriers. Furthermore, the NLRP1 gene variant (rs12150220) demonstrated highly significant expression changes (*p* = 6.17^−3^) in the esophagus (Figure 8).

For NR candidate genes, Figure 5 shows the protein interaction gene network from the STRING. Enrichment analysis of the biological functions and pathways indicates their role in cytokine processes and pathways, which include IL-20 and IL-12. They play a key role in the non-response status by triggering inflammation in the target tissues through their aberrant expressions. The non-responder groups’ genes, by interacting with the key partners, may contribute to hindering the response to VDZ, leading to the severity of the disease symptoms.

**Figure 9:**
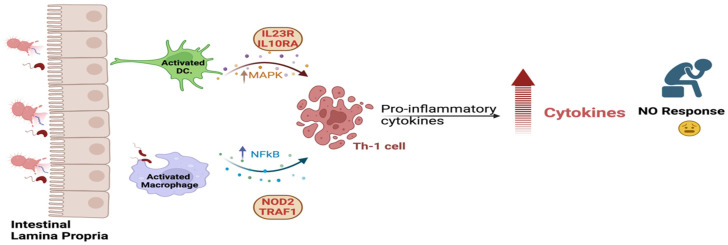
Biological functional roles of candidate genes in VDZ non-responder group.

The biological functions and processes of the non-responder genes’ network are shown in Figure 9. Functional and pathway enrichment of these genes reveals that the IL23R and IL10RA activate the dendritic cells, triggering the MAPK signaling activity. This activation stimulates the Th-1 cell to produce pro-inflammatory cytokines, which upregulate the cytokine secretion. At the same time, NOD2 and TRAF1 enhance the NFkB singling cascade through the activation of macrophages; this will induce the Th-1 cells to produce more pro-inflammatory cytokines. The non-responsive status, in this case, is due to the aggregation of pro-inflammatory cytokines that counteract the inhibitory action of VDZ.

**Figure 10:**
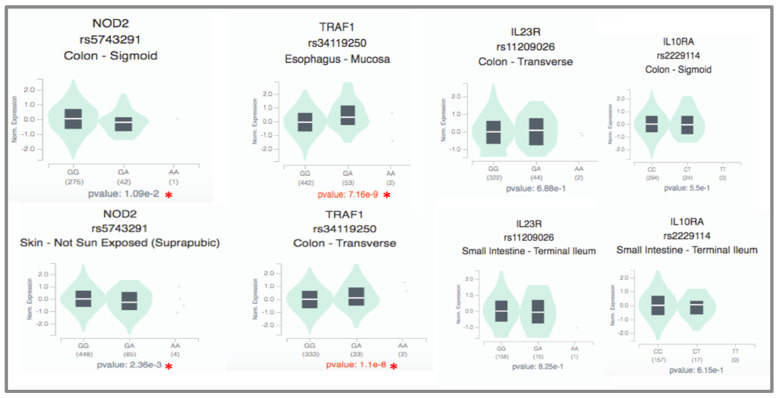
Genotype-Tissue Expression (GTEx) of non-responder genes. * denotes statistically significantly different gene expression recorded in specific tissues between the carriers of the rare variant and common alleles.

To further validate the biological pathways of the candidate genes in the non-responder group, GTEx profiles in a variety of tissues were searched for in the gene variants. We observed a significant change in the expression of the TRAF1 gene with the rare variant (rs34119250) in the esophagus and sigmoid colon (*p* = 7.16^−9^ and 2.88^−5^, respectively). Additionally, with the NOD2 gene variant (rs5743291), a significant change in expression was observed in the sigmoid colon (*p* = 1.09^−2^) and skin *p* = 2.36^−3^) (see Figure 10). IL23R and IL10RA did not show any significant changes in their expression between rare and common variant carriers in any tissues analyzed.

The global population’s MAF is changing rapidly due to the increasing number of samples from various ethnic groups that have been added to the variant databases, such as the RGC Million Exome db, the largest genomic database. All candidate gene rare variants were searched for in the pooled Middle Eastern Arab data along with global MAF data and are presented in Table 8. This reveals differences in variant frequencies in Arabs when compared to other ethnic groups; for example, in the IL27 variant—low, the IL4 variant—high, and the NLRP1 variant—very high (approximately 9 out of 100 Arabs have this variant, when compared to approximately 2.4 in 1 million from RGC).

## 4. Discussion

Most genetic studies on IBD have largely concentrated on identifying the common disease susceptibility variants with small effect sizes through GWASs in Caucasians [25]. However, rare and highly penetrant variations identified through population-specific cohorts or family-focused research have immense potential to catch the variants with high effect sizes on complex diseases such as IBD [17,26]. Many large-scale sporadic case-control studies on WES-based rare variant burden analysis (RVB) have previously identified several strong risk loci for complex diseases, such as schizophrenia [27], Alzheimer’s disease [28], autism [29], and CD [30]. According to a recent systemic review and meta-analysis of IBD in the Arab world, the consanguinity rate in Saudi Arabian IBD patients is high, 57.6%, which is similar to that in the general population as well [4,31]. The present study, by a novel approach, utilized the exome data from highly consanguineous Saudi Arabian ABD patients with biological functional enrichment with a range of bioinformatic tools and databases to identify the unique genetic signature for VDZ drug response.

This study utilized the power of large-scale multi-domain computational biological databases and tools to prioritize the rare coding variants in genes and key disease pathways, aiming to facilitate personalized genomic analysis of patients. This study prioritized NOD2, IL23, IL10, IL27, TRAF1, CARD9, TYK2, IL4, and NLRP1 genes with rare variants from non-responders and responders, respectively, as potential candidate biomarkers for VDZ drug response. A stringent filtration for allele frequency, biological functions, and genetic evidence resulted in 261 and 230 genes in responders and non-responders, respectively. Genes prioritized by GWASs that overlap in both responder and non-responder groups play a crucial role in the etiology of IBD through the inflammatory pathway [32,33] and cytokine interaction process [34,35,36]. The network interaction of non-responder potential candidate genes showed many cytokines that play a role in IBD pathogenesis. IL-20 and IL-12RB1 with the co-expression of IL-12 is a key regulator of the inflammatory cascade of non-responder genes, confirming a previous study [37]. This demonstrates a clear role for *IL-12* in the initiation of intestinal inflammation, disrupting the epithelial barrier. IL23, on the other hand, is important in chronic intestinal inflammation [38]. Moreover, most of the identified non-responder potential predictive markers are related to pro-inflammatory cytokines/chemokines or their receptors (NOD2, IL23R, TRAF1, IL10RA, and IL27) [32]. The majority of these genes interact with IL12A. The activity of IL23 is dependent on IL12Rβ1, a type 1 transmembrane receptor that physically associates with the p40 domain common to both IL12 and IL23 and promotes their respective signaling pathways [39]. All the previous studies identified the critical role of NOD2, IL23, IL10RA, and TRAF1 as risk factors for IBD in GW studies and in animal models for UC or CD [40,41,42].

Most NR genes act on pro-inflammatory cytokine secretion; for example, *NOD2* activates NFκB and MAPK, and, thus, the transcription of pro-inflammatory molecules such as IL6, IL8, IL1β, and TNF-α, which alter Paneth cells’ ability to recognize and eliminate invading pathogens that cause the development of inflammatory bowel lesions [43,44]. Also, TRAF1 regulates both canonical and non-canonical *NFκB* pathways as well as MAP kinase activation, and influences pro-inflammatory cytokine production and inflammatory responses [42,45]. This upregulation of pro-inflammatory cytokines may potentially hinder the VDZ in inhibiting the alpha 4 beta 7 integrin. Two prior meta-analyses have examined NOD2/CARD15 polymorphisms in CD to confirm the non-responder role of NOD2 in IBD therapy [41,46]. Moreover, a few animal models support the role of *NOD2* and TRAF1 in the development of CD [47,48]. In addition, interleukin-23 (*IL23*) is a pro-inflammatory cytokine, produced primarily by macrophages and antigen-presenting cells (APCs) after antigen stimulation. Studies conducted in both mice and humans have shown that IL23 acts as a major pathogenic pro-inflammatory factor in IBD [49,50]. Targeting the IL23 pathway is an important opportunity for drug development. Multiple lines of evidence support the IL23 blockade as a therapeutic strategy for IBD [51]. In a mouse model, an IL23 blockade suppressed T cell-mediated colitis and reduced inflammation to a greater extent than an IL12 blockade alone. Ustekinumab is a monoclonal antibody that targets the shared p40 subunit of IL-12/23 and is approved for the treatment of moderate-to-severe CD and UC [51].

The responder genes enhance the drug action of VDZ by its anti-inflammatory/immune-modulatory effect [43,52,53]. Moreover, most of the key interactors of responder genes through IL13RA1 act as pleiotropic cytokines in the activation of IL4 to induce inhibitory responses, which contribute to inflammatory diseases [54]. Anti-inflammatory cytokines play a crucial role in controlling pro-inflammatory cytokine production. This investigation identified rare variants in IL-4 in more responders than in non-responders. A possible explanation for this might be that it is contributing to the enhanced production/action of anti-inflammatory cytokines in IBD by VDZ [55]. Previous studies confirm that IL-4 acts as an anti-inflammatory and immunomodulatory factor by suppressing the pro-inflammatory IL1, IL6, IL12, and TNF-alpha secretion [56]. IL4 promotes the healing processes accomplished by macrophages and has an alleviating effect upon patients with colitis [57]. TYK2 is involved in the signaling pathway of various cytokines, including IL4, which are responsible for transmitting signals from cytokine receptors to the nucleus. Therefore, TYK2 is necessary for IL4 to exert its effects on immune cells. In an animal model, the administration of the antagonist of IL4 receptorα for the treatment of atopic dermatitis has caused enteritis as a side effect [58]. These findings lead to the assumption that the immune response mediated by Th2 cells may act as a protective factor against VDZ IBD. In addition, CARD9 (Caspase Recruitment Domain 9) plays a role in the response to bacteria by interacting with NOD2 [59]. The previous studies reported the protective role of *CARD9* S12NΔ11 (with the deletion of exon 11) as a protective variant. This allele truncates the CARD9 C-terminus of the protein to provide strong protection against IBD. Those individuals with CARD9 S12NΔ11 have decreased TNF-α and IL-6 production [60]. Rare variants of NLRP1 in the responders may attenuate gastrointestinal inflammation. Knock-out *Nlrp1b*(−/−) mice demonstrated significant increases in morbidity, inflammation, and tumorigenesis [53]. Taken together, these data identify NLRP1 as an essential mediator of the host immune response during IBD and cancer. These findings are consistent with a model whereby multiple NLR inflammasomes attenuate disease pathobiology through modulating IL-1β and IL18 levels in the colon [53]. In the current study, rare variants of these genes among the responders highlight potential biomarkers for better VDZ response. The current study results emphasize the importance of the early prediction of drug response, which is essential to prevent disease resistance and damage to the intestines in drug-resistant and poorly responding patients.

We acknowledge some limitations of this study. First, this study involved a small sample size, which limits the robustness of the findings. The recruitment of more patients could help in validating the potential candidate genes identified in this study. The main reasons for the small sample size in this study are (a) the poor response of patients in joining as participants in this research project in general, especially during and after the pandemic, due to the restricted movement of the population; (b) the cost of the WES processing is high; and (c) a limited number of patients were allowed to be treated with this drug due to limited hospital funds and stringent patient selection for this costly treatment regimen in Saudi Arabia. Second, a lack of large-scale population-specific genomic variant databases in the Arab world in the public domain restricted us in confirming the minor allele frequency in the local Arab population, which is genetically unique. Last, a lack of large-scale GWASs in the genetically unique Arab populations has also contributed to the sparse knowledge of the genetic susceptibility to complex diseases, including IBD. The lack of laboratory-based validation in many IBD patients treated with VDZ is also a limitation of this study.

## 5. Conclusions

This is the first study to explore WES data for IBD drug response in the Middle Eastern Arab region. More than a million variants across the exomes of the 16 IBD patients were searched for and analyzed with a variety of advanced bioinformatic tools for pathogenicity prediction, population frequencies, protein–protein interaction networks, pathways, functional enrichment, etc., and animal models were also analyzed. This study highlights the potential critical genetic signature of Saudi Arabian IBD patient response/non-response status to VDZ. The present investigation lays the foundation to integrate WES with extensive integrated multi-omics data analysis to identify drug response candidate genes for chronic complex diseases in Saudi Arabia. Future research should look for rare variant burdens in the target genes and other critical pathway genes in large sample sizes for more validation. This will have the positive downstream effect of improving drug adherence. Moreover, this will help clinical management teams and the government cover more IBD patients nationwide who are predicted to be responders to the drug and reduce the economic burden of patient care. Predicting responders early will spur the rapid introduction of an efficient personalized medical management program for IBD across the country.

## Figures and Tables

**Table 1:** Inclusion/exclusion criteria to identify and to recruit the participants.

Inclusion Criteria	Exclusion Criteria
Aged between 18 and 80 years; male or female Saudi patients; capable of giving voluntary informed consent; diagnosis of moderate-to-severe active UC/CD (Mayo endoscopic sub-score > 1 for UC OR presence of frank ulcerations for CD); and patients naïve to VDZ.	Signs of abdominal abscess; any colonic resection; either total or subtotal colectomy; known fixed stenosis of the intestine, ileostomy, or colostomy; use of non-permitted IBD therapies within 30 or 60 days; active or dormant TB infection; hepatitis C or B chronic infection; malignancy; small or large bowel malignancy; being incapable of complying with study protocols or attending study visits.

**Table 2:** Databases used (including version) for the variant identification and annotation.

Software	Version
Mapping Reference	Hg38 (original GRCh38-NCBI, Dec.2013).
dbSNP	154
1000Genome	Phase3
Clinvar	07/2021
ESP	ESP6500SI_V2
dbNSFP	dbNSFPv4.2c

**Table 3:** Computational tools used to curate the exome sequence and annotate the variants.

Software	Version
BWA	Bwa-0.7.17
Picard	Picard-tools-2.18.2-SNAPSHOT
GATK	GATKv4.0.5.1
SnpEff	SnpEff 5.0e 9 March 2021

**Table 4:** Examples of some of the complex biological databases with extensive bioinformatic output for complex queries used.

Evidence Tool	Outcomes
Open Target Platform	This website provides access to multiple computational tools and databases that aid in identifying the causal or functional annotations between the clinical phenotype and drug target genes [20].
TOPPFun	The ToppGene platform is used to enrich a group of genes based on function and cellular localization, pathways, etc., to prioritize candidate genes by extensively using an array of protein interaction and functional annotations network datasets [21].
STRING database	This protein–protein interaction database encompass both physical and functional connections of all genes [22].
Genotype-Tissue Expression (GTEx)	Human gene expression data resource from various tissues and organs used to correlate their relationships to genetic variation [23].
RGC	New whole-exome/genome database from more than a million people of different ethnicities in the public domain for minor allele frequency of variants to identify the rare variants in the world population [24].

**Table 5:** Total rare variants in responder and non-responder groups based on MAF. (Range: extremely rare <0.01; very rare 0.01–0.0299; and rare 0.03–0.05).

	Variant Prioritizations		
Group	Extreme Rare	Very Rare	Rare
R	2590	3308	9276
NR	1808	2421	5839

**Table 8:** Alleles’ frequency in Middle Eastern Arabs compared to other populations in non-responder and responder variants.

Gene/SNP	MAF	1000 G	GnomAD	RGC—All	RGC—Arab	Saudi MAF
Non-Responder						
NOD2	rs5743291	0.0332	0.063	0.077	0.114	0.0694
IL-23R	rs11209026	0.023	0.042	0.055	0.057	0.0486
IL-10RA	rs2229114	0.017	0.032	0.040	0.040	0.0312
IL-27	rs147413292	0.020	0.050	0.053	0.058	0.0347
TRAF1	rs34119250	0.034	0.051	0.060	0.062	0.0694
Responder						
CARD9	rs114895119	0.002	0.004	0.004	0.009	0.0035
TYK2	rs2304255	0.044	0.062	0.066	0.071	0.0417
IL-4	rs2234895	0.047	0.069	0.077	0.112	0.1146
NLRP1	rs11653832	-	0.00000119	0.000002433	0	0.0938

## Data Availability

Data that support the findings of this study are available from the corresponding author upon request.

## Supplementary Materials

The supporting information can be downloaded at https://www.mdpi.com/article/10.3390/biomedicines13020459/s1. Informed consent, and Data capture Sheets.

## Abbreviations

The following abbreviations are used in this manuscript:

IBD	inflammatory bowel disease
MACAM-1	mucosal vascular addressin cell adhesion molecule
KSA	Kingdom of Saudi Arabia
VDZ	vedolizumab
