# Supplementary material for: High-Throughput Whole-Exome Sequencing and Large-Scale Computational Analysis to Identify the Genetic Biomarkers to Predict the Vedolizumab Response Status in Inflammatory Bowel Disease Patients from Saudi Arabia
Source: Biomedicines. 2025 Feb 13;13(2):459. doi: 10.3390/biomedicines13020459 (PMC11852680; doi:10.3390/biomedicines13020459)
Supplement: Supplementary file 1 [file biomedicines-13-00459-s001.zip › biomedicines-3459948-supplementary.pdf]

Informed Consent

You are invited by (Prof. Samir bin Ayda Al-Harthi - Professor in the Department of Clinical Pharmacology at the College of Medicine

Request to participate in a scientific research entitled:
Inflammatory Bowel Disease Precision Medicine Initiative in the Kingdom of Saudi Arabia An integrated pharmacokinetic study of biomarkers for early identification of responders to Vedolizumab

Research information

Inflammatory bowel disease is a chronic and acute inflammatory disease that affects any area of the digestive system. This disease is characterized by an increase in its occurrence over time and requires strict treatment until the severity of symptoms decreases and the quality of life improves. Although in recent years there has been a major development in the method of treatment as it has changed from the use of initial treatments to the use of biological treatments that target inflammation and its accumulation sites, there is still a number of patients who do not respond to any type of treatment. Recent studies have proven that there are a number of reasons that lead to non-response, for example, antibodies may form against the drug that reduce the effectiveness of the drug, or there may be a type of protein that is abundant in inflammation that the drug was unable to Inhibiting it. Finally. There may be a genetic factor in the patient that prevents response. In this study, we will explore the reasons leading to non-response to Vedolizumab so that we can form a database of patients with inflammatory bowel disease in Saudi Arabia, which doctors can rely on and treat each patient independently without exposing the patient to the side effects of other medications. And even reduce the number of times the patient is hospitalized and increases the survival rate.

This research is being conducted at King Abdulaziz University Hospital and a blood sample (2-4 ml) will be taken at each visit to take the dose of VDZ over a period of 0,2,6 weeks

Consent of the human participant:

Signature:

أنت مدعو/ أنت مدعوة من قبل (بروف. سمير بن عيضة الحارثي-أستاذ بقسم علم الأدوية بكلية الطب) إلى المشاركة في بحث علمي تحت عنوان:
مبادرة الطب الدقيق لمرض التهاب الأمعاء في المملكة العربية السعودية: بحث حركي دوائي متكامل عن المؤشرات الحيوية للتعرف المبكر على المستجيبين لفيدوليزوماب

معلومات البحث

مرض الأمعاء الإلتهابية هو مرض إلتهابي مزمن وحاد يصيب أي منطقة في الجهاز الهضمي. هذا المرض يتصف بإزدياد حدته مع مرور الوقت ويحتاج إلى علاج صارم حتى تخف حدة الأعراض وتتحسن جودة الحياة. على الرغم من أنه في السنوات الأخيرة كان هناك تطور كبير في طريقة العلاج حيث أنه تغير من إستخدام العلاجات المبدئية إلى إستخدام علاجات بيولوجية تستهدف الالتهابات وأماكن تجمعها، إلا أنه لا زال عدد من المرضى لا يستجيبون لأي نوع من العلاج. الدراسات الحديثة أثبتت أن هناك عدد من الأسباب التي تؤدي إلى عدم الإستجابة، فمثلا من الممكن أن تتكون أجسام مضادة ضد الدواء تقلل من فعالية الدواء، او من الممكن أن يكون هناك نوع من البروتينات التي تتواجد بكثرة في الإلتهابات لم يتمكن الدواء من تثبيطها. وأخيرا، من الممكن أن يكون هناك عامل وراثي للمريض يمنع من الإستجابة. نحن في هذه الدراسة سوف نقوم بإستكشاف الأسباب المؤدية إلى عدم الإستجابة لدواء الفيدوليزوماب حتى نتمكن من تكوين قاعدة بيانات لمرضى الأمعاء الإلتهابية في السعودية، ويستند عليها الأطباء ويتمكنوا من علاج كل مريض بصفة مستقلة من غير تعريض المريض للآثار الجانبية للأدوية الأخرى، وحتى نقلل من عدد مرات التنويم للمريض داخل المستشفى ونزيد من نسبة النجاة.

يجري هذا البحث في مستشفى جامعة الملك عبد العزيز وسوف يتم أخذ عينة دم (٢-٤ مل) عند كل زيارة لأخذ جرعة الفيدوليزوماب على مدى ٠,٢,٦ أسابيع.

موافقة المشارك/ الإنسان: **التوقيع:**

Patient data sheet (Ulcerative colitis)

Name		Age		Sex		Nationality	
File number		Vedolizumab start date					
Vedolizumab treatment assignment	0-weeks ☐	2-weeks ☐	6-weeks ☐				
Exposure to Anti-TNF	Naïve ☐	Exposed ☐					
If patient exposed	Name of agent: ________________<br>Duration: ________________<br>Response: ________________						
Patient reported outcome	________________<br>________________<br>________________<br>________________<br>________________<br>________________						
Family history	________________<br>________________<br>________________<br>________________<br>________________						
IBD history	Age at diagnosis<br>________<br>Disease Severity<br>________________<br>________________<br>________________<br>________________<br>Pervious hospitalization<br>________________<br>________________<br>________________<br>________________<br>________________<br>________________<br>________________<br>________________<br>________________						

	____<br><br>Extra intestinal manifestations<br><br>Gastritis<br>Toxic megacolon<br>Dehydration<br>Hypotension<br>Altered consciousness<br>Electrolyte abnormalities
Physical examination	Ht. [ ] Wt. [ ]<br>T. [ ] BL.P. [ ] R.R. [ ] H.R. [ ]
Assessment of UC	Partial Mayo clinical score<br><br>Frequency of bowel movement<br>0=Normal for the patient<br>1=1-2 stools per day in addition to usual<br>2=3-4 stools per day in addition to usual<br>3>-5per day beyond the usual<br><br>Rectal bleeding<br>0=No bleeding<br>1=Blood streaks in less than half of evacuations<br>2=Evidence of fresh blood in most of the evacuation<br>3=Bowel movements with fresh blood<br><br>PGA/Physician assessment disease activity<br>0=Normal<br>1=Mild<br>2=Moderate<br>3=Severe<br><br>Endoscopic findings<br>0=Normal mucosa or inactive disease<br>1=Mild disease (erythema, loss of vascular pattern, mild friability)<br>2=Moderate disease (obvious erythema, loss of vascular pattern, friability, erosions)<br>3=Severe disease (spontaneous bleeding, ulceration)

Score (points)	Disease severity
≤2 and no subscore>1	Clinical remission
3-5	Mild activity
6-10	Moderate activity
11-12	Severe activity

Lab work	-Fecal calprotectin at 0 weeks__________, at 6 weeks___________<br>-CRP__________________<br>-Albumin_____________<br>-Hemoglobin__________

Patient data sheet (Crohn's disease)

Name		Age		Nationality		Sex	
File No.		Vedolizumab start date					
Vedolizumab treatment assignment	0-weeks ☐		2-weeks ☐		6-weeks ☐		
Anti-TNF Exposure	Naïve ☐			Exposed ☐			
If patient exposed	Name of agent: ______<br>Duration: ______<br>Response: ______						
Patient reported outcome (PRO'S)	______<br>______<br>______<br>______<br>______<br>______<br>______<br>______<br>______<br>______<br>______<br>______						
Family History	______<br>______<br>______<br>______<br>______<br>______<br>______<br>______						

IBD history	Age at diagnosis<br>______________________________<br><br>Disease severity<br><br>____<br>____<br>____<br>____<br>____<br>____<br>____<br>____<br>____________<br><br>Pervious hospitalization/pervious surgeries<br>____<br>____<br>____<br>____<br>____<br>____<br>____<br>____<br><br>Extra-intestinal manifestation:<br>-Arthritis or arthralgia ☐<br>-Uveitis/iritis ☐<br>-Erythema nodosum, pyoderma gangrenosum, or aphthous stomatitis ☐<br>-Anal fissure, fistula, or abscess ☐<br>-Temperature over 37.8°C in the last week ☐<br>-Finding of an Abdominal Mass ☐<br>-Anemia and Weight Change ☐
Physical Examination	Height ☐ Weight ☐<br>T ☐ RR ☐ H.R. ☐ BP ☐

Assessment of CD (CDAI)	Items	Score
	Patient Reported Stool Pattern	
	Average number of liquid or soft stools per day over 7 days	14 points per stool
	Using diphenoxylate or loperamide for diarrhea	30 points
	Average Abdominal Pain Rating Over 7 days	
	None	0
	Mild pain	35
	Moderate pain	70
	Severe pain	105
	General Wellbeing Each Day Over 7 days	
	Well	0
	Slightly below average	49
	Poor	98
	Very poor	147
	Terrible	196
	**Complications**	
	Arthritis or arthralgia	20
	Iritis or uveitis	20
	Erythema nodosum, pyoderma gangrenosum, or aphthous stomatitis	20
	Anal fissure, fistula, or abscess	20
	Other fistula	20
	Temperature over 37.8°C in the last week	20
	Finding of an Abdominal Mass	
	No mass	0
	Possible mass	20
	Definite mass	50
	Anemia and Weight Change	
	Absolute deviation of hematocrit from 47% in males or 42% in females	6 points per percent deviation
	Percentage deviation from standard weight	1 point for each percent deviation
	Total criteria point count	

HBI score

Variable	Scoring
General well being (0-4)	0=very well, 1=slightly below average, 2=poor, 3=very poor, 4=terrible
Abdominal pain (0-3)	0=none, 1=mild, 2=moderate, 3=sever
Abdominal mass (0-3)	0=none, 1=dubious, 2=definite, 3=tender
No. Of liquid stools	1 per occurrence
Extra-intestinal manifestation	1 point for each (Arthralgia, Uveitis, Erythema Nodosum, Pyoderma gangrenosum, Aphthous Ulcer, Anal fissure, New Fistula, Abscess)

HBI score o 4 is considered as remission.
HBI score ≥ 7 is considered as presence of active disease.

Endoscopic score (SES-CD)

Variable	0	1	2	3
Presence of ulcers	None	Aphthous ulcers (0.1-0.5)	Large ulcers (0.5-2)	Very large ulcers (>2)
Ulcerated surface	Unaffected segment	<10%	10-30%	>30%
Affected surface	None	<50%	50-75%	>75%
Presence of narrowing	None	Single, can be passed	Multiple, can be passed	Cannot be passed
Number of affected segments	All variables=0	At least one variable ≥1		

Lab work

-Fecal calprotectin at 0 week ______, at 6 weeks________

-Albumin_________

-CRP___________

-Hemoglobin________
